# Elucidating the mechanisms underlying Alzheimer's disease-associated genetic polymorphisms

**DOI:** 10.1186/1750-1326-7-S1-L12

**Published:** 2012-02-07

**Authors:** Steven Estus

**Affiliations:** 1 Department of Physiology, Sanders-Brown Center on Aging, University of Kentucky, Lexington, KY, USA

## Background

Recent genome-wide association studies (GWAS) have yielded a bounty of single nucleotide polymorphisms (SNP)s related to Alzheimer's disease (AD) or conditions associated with AD. Here, we will present several vignettes that illustrate approaches to use these results to clarify AD-related pathways and potential therapeutics.

## Methods

SNPs of interest are identified from GWAS of AD or AD-related conditions such as cholesterol or rheumatoid arthritis; these latter SNPs are then evaluated for association with AD by using PLINK-mediated logistic regression analysis of AD GWAS data. Resulting AD-associated SNPs are then evaluated further for their impact on gene expression or splicing in human brain autopsy samples by real-time PCR and immunostaining. Candidate SNPs are evaluated for function by using in vitro studies in transfected cells.

## Results

Results will be presented along three fronts. First, SNPs associated with both cholesterol and with AD (other than APOE SNPs) include SNPs within LIPC and HMGCR. In particular, the HMGCR SNP (rs3846662) is associated with the splicing efficiency of HMGCR exon 13; the allele that reduces exon 13 inclusion has been reported to reduce HMGCR activity and is associated with reduced AD risk. This SNP has also been associated with statin response. Overall, these results suggest that this SNP may modulate AD and the response of AD to statin treatment. Second, SNPs associated with rheumatoid arthritis are generally not associated with AD, reinforcing the hypothesis that rheumatoid arthritis is associated with reduced AD risk, perhaps because the associated long-term anti-inflammatory use. Third, the primary AD-associated SNP within CLU, rs11136000, is associated with the expression of CLU1 but not CLU2 in the brain. Both CLU1 and CLU2 produce soluble clusterin protein in transfected cell studies. Since the protective allele of rs11136000 is associated with increased CLU1 expression, agents that upregulate CLU1 may reduce AD risk. Of particular interest, histone deacetylase inhibitors (HDACI) induce CLU1 robustly, suggesting that HDACI such as the widely used epilepsy drug valproic acid may have some utility in reducing AD risk. Lastly, we note that normalizing RNA measurements to correct for variation in cell-type within the brain fragments used for RNA preparation in these studies was critical to discerning SNP association with gene expression or splicing.

## Conclusion

Although AD GWAS results have not yielded another AD risk factor with the impact of the APOE SNPs, they have succeeded in identifying “bottlenecks” in AD-related pathways that modulate AD risks. Hence, elucidating the actions associated with these SNPs by using a combination of bioinformatics, human tissue correlative studies and in vitro functional studies may provide insights into AD therapeutic strategies.

